# Robotic transaxillary thyroidectomy: time to expand indications?

**DOI:** 10.1007/s11701-023-01594-y

**Published:** 2023-04-17

**Authors:** Leonardo Rossi, Andrea De Palma, Lorenzo Fregoli, Piermarco Papini, Carlo Enrico Ambrosini, Chiara Becucci, Benard Gjeloshi, Riccardo Morganti, Puccini Marco, Gabriele Materazzi

**Affiliations:** 1 Department of Surgical, Medical and Molecular Pathology and Critical Area, University of Pisa, Pisa, Italy; 2 Section of Statistics, University of Pisa, Pisa, Italy

**Keywords:** Robotic transaxillary thyroidectomy, Robot-assisted surgery, Remote-access thyroidectomy, Thyroidectomy, Thyroid

## Abstract

In 2016, the American Thyroid Association published a statement on remote-access thyroid surgery claiming that it should be reserved to patients with thyroid nodule ≤ 3 cm, thyroid lobe < 6 cm and without thyroiditis. We retrospectively enrolled all patients who underwent robotic transaxillary thyroidectomy between February 2012 and March 2022. We compared surgical outcomes between patients who presented a thyroid gland with a nodule ≤ 3 cm, thyroid lobe < 6 cm and without thyroiditis (Group A) and patients without these features (Group B). The rate of overall complications resulted comparable (*p* = 0.399), as well as the operative time (*p* = 0.477) and the hospital stay (*p* = 0.305). Moreover, bleeding resulted associated to thyroid nodule > 3 cm (*p* = 0.015), although all bleedings but one occurred in the remote-access site from the axilla to the neck. In experienced hands, robotic transaxillary thyroidectomy is feasible and safe even in patients with large thyroid nodules or thyroiditis.

## Introduction

Since the introduction of robot-assisted gasless transaxillary thyroidectomy (RATT) in South Korea in 2009 [1], several studies reported endearing results regarding its feasibility and safety [2–4]. Nonetheless, they are mostly limited to Asian countries, whereas, after the initial enthusiasm generated from the Korean experience, RATT has lost popularity in Western World, especially in North America where the procedure shifted away from high-volume institutions [5]. Moreover, due to significant differences in terms of body habitus and thyroid pathology between Asian and Western patients, some doubts regarding the applicability of RATT to European and American patients initially raised up.

In this uncertain context, The American Thyroid Association (ATA) Statement on Remote-Access Thyroid Surgery of 2016 acknowledged that the robotic transaxillary approach may be performed by experienced surgeons in selected cases; notwithstanding, RATT was recommended only in patients with well-circumscribed nodule ≤ 3 cm, thyroid lobe < 5 to 6 cm in the largest diameter and absence of thyroiditis [6]. Nonetheless, European high-volume institutions surgeons frequently manage patients with thyroid pathology beyond the ATA statement.

The aim of the present study is to assess surgical outcomes of RATT in a large European population, comparing patients who fulfill the ATA suggestions with those with thyroid features beyond these recommendations.

## Materials and methods

This is a retrospective study performed using a prospectively designed database for data collection and analysis. We enrolled all patients who underwent RATT from February 2012 to March 2022 at the University Hospital of Pisa, Endocrine Surgery Unit. All patients received an extensive explanation of the procedure and provided informed consent. This study had been approved by the internal research board.

All procedures were performed with the da Vinci robotic system, both the SI and the XI versions, using a single axillary incision and three robotic arms. Data regarding patient demographics, thyroid volume, diameter of the largest nodule, extent of surgery, operative time, postoperative length of hospital stay, postoperative complications and histology were recorded. The preoperative evaluation of patients consists of neck ultrasound (NUS), thyroid function test and US-guided fine-needle aspiration cytology if required. We routinely provided counseling about the conduct, risks and alternatives of RATT and conventional thyroidectomy.

Selection criteria for RATT were: sonographically determined nodule diameter ≤ 75 mm for benign nodules and ≤ 35 mm for malignant nodules and expressed preference of the patients for transaxillary approach. Exclusion criteria included previous neck surgery or radiotherapy, locally advanced cancer, Graves’ disease and shoulder or arms disease. The choice of surgery (hemithyroidectomy vs. total thyroidectomy) was in concordance with the American Thyroid Association management guidelines for adult patients with thyroid nodules and differentiated thyroid cancer at the time [7].

Operative time was defined from the skin incision to the skin closure. We defined postoperative hypocalcemia as an albumin-corrected calcium level of < 8.0 mg/dl or inability to interrupt calcium therapy [8]. Recurrent laryngeal nerve (RLN) palsy was diagnosed by an independent laryngologist in case of documented vocal cord mobility alteration at fiberoptic laryngoscopy. We used the cutoff of 6 months to discriminate between transient and permanent postoperative hypocalcemia and RLN palsy. Bleeding was defined as a hemorrhage that could be treated by surgical revision or by conservative management (cervical hematoma). Unconventional complications were defined as complications of novel introduction mainly related to the remote access or to the position of the patient or which rarely occur during conventional thyroidectomy, such as brachial plexus palsy, surgical track seeding, great vessels injury, tracheal injury, skin flap perforation, Bernard-Horner syndrome.

Surgical operations were performed as previously described [9].

Patients were divided into two groups on the basis of the ATA statement on remote-access thyroidectomy [6]: patients who presented a thyroid gland which fulfill the criteria were included in Group A (well-circumscribed nodule ≤ 3 cm, thyroid lobe < 6 cm in the largest diameter and absence of thyroiditis), whereas patients with thyroid out of such features were included in Group B.

The primary endpoint of the study was the overall rate of postoperative complications; secondary endpoints of the study were operative time and length of hospital stay.

### Statistical analysis

Categorical data were described by absolute and relative (%) frequency; continuous data were summarized by mean and standard deviation. Statistical analysis was performed using the Chi-square test for categorical variables and *t* test for continuous variables (SPSS v.28 technology). Significance was fixed at 0.05.

## Results

Between February 2012 and March 2022, 650 RATT were performed at University Hospital of Pisa, Endocrine Surgery Unit. Overall, 650 operations were performed on 614 patients, 610 (99.3%) of which were females, whereas 4 (0.7%) were males. 293 (45.1%) out of 650 cases fulfill the ATA recommendations (Group A), whereas 357 (54.9%) were beyond these criteria (Group B). The two groups resulted comparable in terms of age, body mass index (BMI), type of surgery and histology. The estimated thyroid volume resulted significantly higher in group B (*p* < 0.001). Features of the two groups are summarized in Table 1.

**Table 1:** Demographic, clinical and surgical variables

	Group A (*n* = 293; 45.1%)	Group B (*n* = 357; 54.9%)	Total (*n* = 650)	*p* value
Age (years; mean ± SD)	36.5 ± 10.2	37.1 ± 10.5	36.8 ± 10.3	0.302
BMI (kg/m^2^; mean ± SD)	21.8 ± 2.6	21.5 ± 2.7	21.6 ± 2.7	0.292
Thyroid volume (ml; mean ± SD)	15.6 ± 7.3	21.4 ± 10.6	18.8 ± 9.7	< 0.001
Nodule size (mm; mean ± SD)	17.6 + 7.4	30.5 + 12.7	24.8 + 12.4	< 0.001
Thyroid lobe size (mm; mean ± SD)	43.3 + 7.1	47.8 + 9.3	45.8 + 8.7	< 0.001
Sex, *n* (%)				NA
Male	3 (1.0%)	1 (0.3%)	4 (0.6%)	
Female	290 (99.0%)	356 (99.7%)	646 (99.4%)	
Surgery, *n* (%)				0.217
Hemithyroidectomy	157 (53.6%)	210 (58.8%)	367 (56.5%)	0.137
Thyroidectomy	116 (39.6%)	129 (36.1%)	245 (37.7%)	0.366
Completion of thyroidectomy	19 (6.5%)	17 (4.8%)	36 (5.5%)	0.194
Isthmusectomy	1 (0.3%)	1 (0.3%)	2 (0.3%)	NA
Histology, *n* (%)				0.950
Benign	132 (45.1%)	210 (58.8%)	342 (52.7%)	0.654
Toxic adenoma	2 (0.7%)	4 (1.1%)	6 (0.9%)	0.808
Micro-DTC	44 (15.0%)	40 (11.2%)	84 (12.9%)	0.975
DTC	115 (39.2%)	103 (28.9%)	218 (33.5%)	0.585

*SD* standard deviation, *DTC* differentiated thyroid cancer, *NA* not available

Table 2 describes the operative outcomes of the case series. Group A and Group B resulted comparable in terms of operative time, either for hemithyroidectomy (76.9 ± 23.6 and 80.2 ± 29.8 min, respectively; *p* = 0.225) and total thyroidectomy (95.0 ± 27.8 and 95.5 ± 28.1 min, respectively; *p* = 0.890), hospital stay (2.3 ± 0.8 and 2.3 ± 1.0 days, respectively; *p* = 0.305) and overall complications rate (8.9% and 5.6%, respectively; *p* = 0.399). Moreover, the rate of transient (5.2% and 2.7%, respectively; *p* = 0.455) and permanent (0.7% and 1.4%, respectively; *p* = 0.332) hypocalcemia in patients who underwent total thyroidectomy or completion thyroidectomy resulted comparable between Group A and B. Furthermore, the two groups resulted comparable in terms of rate of transient (2.4% and 0.8%, respectively; *p* = 0.339) and permanent (0% and 0.3%, respectively; *p* = 0.365) RLN palsy, rate of cervical hematoma (2.0% and 0%, respectively; *p* = 0.058), rate of bleeding which requires reoperation (0% and 0.8%, respectively; *p* = 0.116), rate of seroma (0.7% and 1.1%, respectively; *p* = 0.561) and rate of unconventional complications (1.0% and 0.8%, respectively; *p* = 0.808). Specifically, unconventional complications included 2 (0.3%) tracheal injuries (1 in Group A and 1 in Group B), 2 (0.3%) brachial plexus palsy (1 in Group A and 1 in Group B), 1 (0.2%) track seeding in Group B and 1 (0.2%) Bernard-Horner syndrome in Group A. In our case series, only 1 (0.2%) conversion to open thyroidectomy occurred at the beginning of the experience; the patient was included in Group B.

**Table 2:** Comparison of operative outcomes between Group A and Group B

	Group A (*n* = 293; 45.1%)	Group B (*n* = 357; 54.9%)	Total (*n* = 650)	*p* value
Hospital stay (day; mean ± SD)	2.3 ± 0.8	2.3 ± 1.0	2.3 ± 0.9	0.305
Operative time (min; mean ± SD)	84.6 ± 27.3	84.9 ± 30.4	84.8 ± 29.0	0.477
Hemithyroidectomy	76.9 ± 23.6	80.2 ± 29.8	78.0 ± 27.8	0.225
Total thyroidectomy	95.0 ± 27.8	95.5 ± 28.1	95.4 ± 27.5	0.890
Overall postsurgical complications, *n* (%)	26 (8.9%)	20 (5.6%)	46 (7.1%)	0.399
RLN palsy, *n* (%)				
Transient	7 (2.4%)	3 (0.8%)	10 (1.5%)	0.339
Permanent	0	1 (0.3%)	1 (0.1%)	0.365
Hypocalcaemia, *n* (%)^a^				
Transient	7 (5.2%)	4 (2.7%)	11 (3.9%)	0.455
Permanent	1 (0.7%)	2 (1.4%)	3 (1.1%)	0.332
Cervical hematoma, *n* (%)	6 (2.0%)	0	6 (0.9%)	0.058
Bleeding which requires reoperation, *n* (%)	0	3 (0.8%)	3 (0.5%)	0.116
Seroma, *n* (%)	2 (0.7%)	4 (1.1%)	6 (0.9%)	0.561
Unconventional complications, *n* (%)	3 (1.0%)	3 (0.8%)	6 (0.9%)	0.808
Tracheal injury	1 (0.3%)	1 (0.3%)	2 (0.3%)	NA
Brachial plexus injury	1 (0.3%)	1 (0.3%)	2 (0.3%)	NA
Track seeding	0	1 (0.3%)	1 (0.2%)	NA
Bernard-Horner syndrome	1 (0.3%)	0	1 (0.2%)	NA

*SD* standard deviation, *RLN* recurrent laryngeal nerve, *NA* not available

^a^Considering only total thyroidectomies + completion thyroidectomies

162 (24.9%) out of 650 cases resulted affected by thyroiditis. As reported in Table 3, the presence of thyroiditis did not influence surgical outcomes, except for total thyroidectomy operative time which resulted lower (*p* = 0.018).

**Table 3:** Comparison of operative outcomes between patients with or without thyroiditis

	Patients without thyroiditis (*n* = 488; 75.1%)	Patients with thyroiditis (*n* = 162;24.9%)	Total (*n* = 650)	*p* value
Hospital stay (day; mean ± SD)	2.3 ± 0,9	2.2 ± 0.7	2.3 ± 0.9	0.087
Operative time (min; mean ± SD)	85.3 ± 27.5	83.4 ± 33.1	84.8 ± 29.0	0.354
Hemithyroidectomy	78.4 ± 23.8	80.0 ± 37.2	78.0 ± 27.8	0.629
Total thyroidectomy	97.8 ± 28.2	88,1 ± 26,0	95.4 ± 27.5	0.018
Overall postsurgical complications, *n* (%)	37 (7.6%)	9 (5.5%)	46 (7.1%)	0.286
RLN palsy, *n* (%)				
Transient	8 (1.6%)	2 (1.2%)	10 (1.5%)	0.717
Permanent	1 (0.2%)	0	1 (0.1%)	0.564
Hypocalcaemia^a^, *n* (%)				
Transient	9 (4.5%)	2 (2.4%)	11 (3.9%)	0.501
Permanent	2 (1.0%)	1 (1.2%)	3 (1.1%)	0.517
Hematoma, *n* (%)	6 (1.2%)	0	6 (0.9%)	0.156
Bleeding which requires reoperation, *n* (%)	3 (0.6%)	0	3 (0.5%)	0.317
Seroma, *n* (%)	3 (0.6%)	3 (1.9%)	6 (0.9%)	0.154
Unconventional complications, *n* (%)	5 (1.0%)	1 (0.6%)	6 (0.9%)	0.639
Tracheal injury	2 (0.4%)	0	2 (0.3%)	NA
Brachial plexus injury	1 (0.2%)	1 (0.6%)	2 (0.3%)	NA
Track seeding	1 (0.2%)	0	1 (0.2%)	NA
Bernard-Horner	1 (0.2%)	0	1 (0.2%)	NA

*SD* standard deviation, *RLN* recurrent laryngeal nerve, *NA* not available

^a^Considering only total thyroidectomies + completion thyroidectomies

52 (8.0%) out of 650 cases presented a thyroid lobe size ≥ 6 cm. As reported in Table 4, having a thyroid lobe size ≥ 6 cm did not result associated to worse surgical outcomes, nor in terms of operative time, hospital stay and complications.

**Table 4:** Comparison of operative outcomes between patients with thyroid lobe < or ≥ 6 cm

	Lobe size < 6 cm (*n* = 598; 92.0%)	Lobe size ≥ 6 cm (*n* = 52; 8.0%)	Total (*n* = 650)	*p* value
Hospital stay (day; mean ± SD)	2.3 ± 0.9	2.4 ± 0.8	2.3 ± 0.9	0.864
Operative time (min; mean ± SD)	84.1 ± 28.5	93.9 ± 34.0	84.8 ± 29.0	0.452
Hemithyroidectomy	78.1 ± 27.0	91.2 ± 46.1	78.0 ± 27.8	0.179
Total thyroidectomy	94.5 ± 27.6	87.9 ± 10.3	95.4 ± 27.5	0,529
Overall postsurgical complications, *n* (%)	40 (6.7%)	6 (11.5%)	46 (7.1%)	0.920
RLN palsy, *n* (%)				
Transient	9 (1.5%)	1 (1.9%)	10 (1.5%)	0.102
Permanent	1 (0.2%)	0	1 (0.1%)	0.871
Hypocalcaemia^a^, *n* (%)				
Transient	9 (3.5%)	2 (7.1%)	11 (3.9%)	0.571
Permanent	2 (0.8%)	1 (3.6%)	3 (1.1%)	0.860
Hematoma, *n* (%)	6 (1.0%)	0	6 (0.9%)	0.690
Bleeding which requires reoperation, *n* (%)	3 (0.5%)	0	3 (0.5%)	0.778
Seroma, *n* (%)	4 (0.7%)	2 (3.8%)	6 (0.9%)	0.609
Unconventional complications, *n* (%)	6 (1.0%)	0	6 (0.9%)	0,716
Tracheal injury	2 (0.3%)	0	2 (0.3%)	NA
Brachial plexus injury	2 (0.3%)	0	2 (0.3%)	NA
Track seeding	1 (0.2%)	0	1 (0.2%)	NA
Bernard-Horner	1 (0.2%)	0	1 (0.2%)	NA

*SD* standard deviation, *RLN* recurrent laryngeal nerve, *NA* not available

^a^Considering only total thyroidectomies + completion thyroidectomies

205 (31.5%) out of 650 case presented a nodule size > 3 cm. As reported in Table 5, having a nodule > 3 cm resulted associated to a higher rate of bleeding which requires reoperation (*p* = 0.015). 2 (1%) out of the 3 bleedings occurred in the surgical track from the axilla to the neck, whereas only 1 (0.5%) occurred in the thyroid bed. Moreover, a longer operative time, either for hemithyroidectomy and total thyroidectomy (*p* = 0.047 and *p* = 0.043, respectively) was associated to nodule size > 3 cm. No other surgical outcomes differ significantly.

**Table 5:** Comparison of operative outcomes between patients with thyroid nodule ≤ or > 3 cm

	Nodule size ≤ 3 cm (*n* = 445; 68.5%)	Nodule size > 3 cm (*n* = 205; 31.5%)	Total (*n* = 650)	*p* value
Hospital stay (day; mean ± SD)	2.3 ± 0.8	2.3 ± 1.2	2.3 ± 0.9	0.975
Operative time (min; mean ± SD)	83.6 ± 26.5	87.5 ± 33.9	84.8 ± 29.0	0.112
Hemithyroidectomy	76.6 ± 22.6	82.3 ± 32.7	78.0 ± 27.8	0.047
Total thyroidectomy	93.3 ± 27.1	101.7 ± 29.2	95.4 ± 27.5	0.043
Overall postsurgical complications, *n* (%)	31 (7.0%)	15 (7.3%)	46 (7.1%)	0.478
RLN palsy, *n* (%)				
Transient	7 (1.6%)	3 (1.5%)	10 (1.5%)	0.674
Permanent	0	1 (0.5%)	1 (0.1%)	0.161
Hypocalcaemia^a^, *n* (%)				
Transient	9 (4.0%)	2 (3.4%)	11 (3.9%)	0.725
Permanent	2 (0.9%)	1 (1.7%)	3 (1.1%)	0.080
Hematoma, *n* (%)	6 (1.3%)	0	6 (0.9%)	0.374
Bleeding which requires reoperation, *n* (%)	0	3 (1.5%)	3 (0.5%)	0.015
Seroma, *n* (%)	3 (0.7%)	3 (1.5%)	6 (0.9%)	0.398
Unconventional complications, *n* (%)	4 (0.9%)	2 (1.0%)	6 (0.9%)	0.374
Tracheal injury	1 (0.2%)	1 (0.5%)	2 (0.3%)	NA
Brachial plexus injury	2 (0.5%)	0	2 (0.3%)	NA
Track seeding	0	1 (0.5%)	1 (0.2%)	NA
Bernard-Horner	1 (0.2%)	0	1 (0.2%)	NA

*SD* standard deviation, *RLN* recurrent laryngeal nerve, *NA* not available

^a^Considering only total thyroidectomies + completion thyroidectomies

## Discussion

Robotic gasless transaxillary thyroidectomy has emerged as a popular remote-access approach, widely diffused in the Far East. In Asian countries, RATT has been shown to be safe with comparable operative outcomes to open thyroidectomy and resulted associated to excellent cosmesis and reduced postoperative pain [10, 11].

Nonetheless, in U.S. after initial interest for the technique, the enthusiasm was dampened after withdrawal of industrial support [5]. Indeed, the company had not obtained a proper U.S. Food and Drug Administration (FDA) approval to market its product for RATT and in 2011 Intuitive Surgical, Inc., stated that they would no longer provide or facilitate any promotion, training, case observations, proctoring or on-site procedural support for use of the da Vinci System in conjunction with thyroidectomy procedures [5, 12]. The announcement leads to a progressively decreased interest for robotic thyroidectomy and a significant drop in RATT volume was observed across U.S. institutions with a fell by more than 50% in 2012 [5]. In that year, Perrier published an editorial to explain why she and his team abandoned performing RATT [13]: the elevated costs, increased operative time, the complexity of set-up progressively tempered the enthusiasm for this approach. Moreover, the Author reported that patients with thyroid cancer, Graves’ disease, large nodules and thyroiditis are not appropriate candidate for robotic thyroidectomy and concluded that although RATT is technically feasible, it should not be advised to be performed [13].

Hinson et al. published in 2015 an analysis of trends of RATT in U.S. [5]. The Authors reported that it has never account for more than 1% of thyroid surgeries per year and that the majority of procedures were performed by low-volume institutions, although these were associated to a significant higher rate of surgical complications compared to high-volume ones (*p* = 0.02) [5]. Moreover, the introduction on novel complications [14] and prohibitive costs hinder the diffusion of RATT in the Western World and several Authors heavily criticized this new approach [15–17].

Furthermore, differences between Eastern and Western patients in terms of body habitus, cultural issues, funding reimbursement and thyroid features raised issues regarding the adoption of this technique in U.S. and Europe. In particular, wide differences emerged regarding the thyroid features of Asian and American patients, mainly related to different approach of the health care systems to thyroid disease and the Korean screening program for thyroid cancer [5, 18]. Accordingly, Kim and colleagues reported in 2018 their experience regarding the first 5000 cases of RATT: 96% of patients were affected by differentiated thyroid cancer (DTC) with a mean tumor size of 0.8 cm [4]; on the other hand, Lin et al. in a North American population study reported a median thyroid nodule size of 2.9 cm and a median thyroid lobe size of 5.4 cm [15]. Similar findings were reported by other Authors [19, 20].

The American Thyroid Association Statement on remote-access thyroid surgery, published in 2016 by Berber et al. [6], underlined the differences between the Korean and American experience with RATT. Moreover, the Authors suggested that this approach can be performed in selected cases but only in high-volume institutions. In particular, they recommended to limit RATT to patients with the following features of the thyroid gland: well-circumscribed nodule ≤ 3 cm, thyroid lobe < 5 to 6 cm in the largest diameter and absence of thyroiditis [6].

Although anthropometrics’ and thyroid’s features of European patients are similar to those of Americans, in Europe some high-volume institutions perform RATT routinary reporting case series of hundreds of patients [14, 21, 22].

We started performing RATT in 2012. At the beginning of our experience there were no accepted guidelines for preoperative patient selection for robotic procedures [23]. Considering the prevalence of multinodular goiter and large thyroid nodules in Italy, we started progressively handling with the robotic system large thyroids, regardless from the presence of thyroiditis. In 2016, when the American Thyroid Association published its statement regarding remote-access thyroidectomy [6], we had already performed more than 200 cases [9], most of them beyond American recommendations. Although we already documented the overall feasibility and safety of RATT reporting excellent complications rate [14], we felt the necessity to further investigate whether our performances in the daily practice with patients beyond ATA recommendations are actually associated to worse surgical outcomes.

Our experience showed no statistically significant differences in terms of morbidity between cases in accordance to the ATA statement on remote-access thyroidectomy and those not. In particular, the overall complication rate (*p* = 0.399), the rate of transient and permanent hypocalcemia (*p* = 0.455 and 0.332, respectively), transient and permanent RLN palsy (*p* = 0.339 and 0.365, respectively), cervical hematoma (*p* = 0.058), bleeding which requires reoperation (*p* = 0.116), seroma (*p* = 0.561) and other unconventional complications (*p* = 0.808) resulted comparable. Moreover, we stratified patients on the basis of each ATA statement parameter and found no statistically significant differences between patients with or without thyroiditis and patients with thyroid lobe < or ≥ 6 cm. Regarding nodule size, no statistically significant differences were documented between patients with nodule ≤ or > 3 cm, except for bleeding which requires reoperation which resulted higher for nodules > 3 cm. Nonetheless, all bleedings but one occurred in the subcutaneous flap from the axilla to the neck, thus without correlation to the thyroid gland.

Our findings mirror previous studies. In particular, Stang et al. in a large retrospective analysis of 302 RATT performed on a North American population reported excellent results in terms of surgical outcomes with low complication rate, with more than half patients discharged on the operation day [24]. Besides, the Authors reported that the complications rate did not differ for clinical variables such as nodule size ≥ 3 cm and thyroiditis. Moreover, Aidan et al. published their experience including large thyroid nodules (average largest diameter of the lesion removed was 3.72 cm) with good outcomes; the Authors included patients with malignant lesions up to 4 cm and benign lesions up to 6 cm [25]. Similarly, Piccoli et al. in a large study from Italy on 449 cases documented excellent results including benign lesions up to 5 cm (median tumor diameter of the case series 2.6 cm) [21]. Furthermore, the Authors did not find nodule size as a factor which influence complications risk, as well as BMI and age.

Regarding operative time, our case series reported optimal results compared to previously published papers [4, 21], although quite far from the target of 68 min proposed by Cabot et al. [26] for the cost equivalence with conventional thyroidectomy. Furthermore, our analysis documented that nodule size > 3 cm resulted associated to longer operations, either considering hemithyroidectomy and total thyroidectomy. This finding is in accordance to Piccoli et al. [21] which reported higher operation time related to tumor diameter. Nonetheless, although statistically significant, in our experience the differences seem not clinically significant both for hemithyroidectomy and total thyroidectomy: indeed, robotic procedures for large thyroid nodules last only 6 and 8 min longer, respectively. Moreover, it has been shown that the trend to a longer operative time for patients with large nodule size is not significant [27] with little effect on the duration of the operation, and this parameter should not hinder the adoption of this approach.

Besides, in our study total thyroidectomy in patients without thyroiditis resulted significantly longer of about 9 min, although this result doesn’t seem to present a real clinical implication.

On contrast with our findings, Son and Colleagues [28] previously reported that thyroid size influence operative time: indeed, handling a thyroid lobe in a narrow surgical space can be challenging and a distance of at least 1 cm between the retractor and the thyroid gland should be assured; moreover, thyroiditis was found as another thyroid-related contributing factor which increased operative time and blood loss. Indeed, thyroiditis is often associated to nodularity, adherence to surrounding tissues and increasing vascularization, making the dissection more difficulty [28, 29]. On the other hand, tumor size was not associated to increased operative time [28].

In 2017, Song et al. [30] scrutinized factors affecting operative time in robotic thyroidectomy and reported that BMI ≥ 23 kg/m^2^ and central compartment dissection were both independent risk factor for prolonged operative time. Moreover, Kandil et al. [20] reported longer operative time in obese patients, either considering the flap creation and console time. Accordingly, Axente et al. [31] and Lee et al. [32] reported an increase in dissection time and console time directly proportional to the patients’ BMI, although these findings are still debated and not reach an unanimous consent [21].

Histological examination of our case series documented 294 patients affected by differentiated thyroid carcinomas (including 8 patients who presented incidental DTC at completion thyroidectomy). Overall, after a mean follow-up of 54.2 ± 30.6 months, all patients are free from structural recurrence at ultrasound examination. Moreover, considering only patients who underwent total thyroidectomy and completion thyroidectomy, after excluding 21.9% of patients lost at follow-up, 66.2% of patients underwent radioactive iodine (RAI) therapy. Furthermore, thyroglobulin (Tg) levels resulted < 0.2 ng/ml in 68.2% of patients, between 0.2 and 1 ng/ml in 20.4% of patients and ≥ 1 ng/ml in 11.5% of patients. Besides, the mean Tg level in patients who underwent RAI therapy was 0.3 ± 0.7 ng/ml, whereas it was 0.9 ± 1.6 ng/ml in patients who underwent thyroidectomy alone.

In 2018 we already described our results on differentiated thyroid carcinomas proving RATT as oncologically safe [9]. Moreover, our results are in accordance to previous studies [2, 4, 33] which demonstrated RATT as safe and feasible even in case of malignant nodules in properly selected cases. Besides, some encouraging results are emerging regarding robot-assisted lateral neck dissection, although almost limited to the Korean experience [34].

Overall, our study demonstrated the feasibility and safety of RATT in patients with large thyroid nodules and thyroiditis, making this approach applicable to a large portion of the Western population, on contrast of Minimally-Invasive Video-Assisted Thyroidectomy which is reserved to a minority of patients [11, 35]. Nonetheless, these data come from a high-volume institution with surgeons highly experienced in endocrine surgery (more than 3000 cases per year) with a huge background of minimally-invasive and robotic procedures; thus, our findings may not be reproducible in all centers. In this context, handling thyroid gland affected by thyroiditis was not associated to worse operative outcomes; furthermore, the nodule size doesn’t represent a limitation for the technique, although associated to a little increase in operative time. On contrast, it is more important to assess the shape of the thyroid and the position of the nodule, rather than the diameter. Thyroid with substernal goiter, as well as nodules located at the upper pedicle or posteriorly (especially in the contralateral lobe) may increase the difficulty of the operation and impact on postoperative outcomes. Patients candidate to RATT must undergo cervical ultrasound preoperatively to evaluate accurately these aspects which potentially may lead to exclude them from the possibility of undergoing RATT, even if affected by small nodules without thyroiditis. Last but not least, the team training is of primary importance: during RATT, an experienced team at the operative table facilitates tremendously the surgeon, either with surgical actions and with the docking of the robot. Taking everything into consideration, according to our experience and to previous studies [9, 21, 36], we believe that an initial learning curve of 20 lobectomies and 40–50 total thyroidectomies are required to start approaching more difficult cases beyond ATA recommendations.

This study harbors limitations. First of all, the retrospective nature of the study may have led to selection bias. Moreover, we did not assess the oncologic outcomes of groups since the study was focused only on surgical outcomes. Third, this series was performed at a single institution, thereby limiting the generalizability of the results.

All in all, in high-volume institutions RATT is feasible even in cases beyond ATA recommendations [6]. Nonetheless, we suggest to follow these indications at the beginning of the experience to select ideal cases, such as women with a normal BMI and small nodule without thyroiditis, scheduled to undergo hemithyroidectomy.

## Data Availability

Data available upon reasonable request.
